# Treatment Results of Geotropic and Apogeotropic Horizontal Canal Benign Paroxysmal Positional Vertigo in a Tertiary Dizziness Clinic

**DOI:** 10.3389/fneur.2021.720444

**Published:** 2021-07-19

**Authors:** Britta D. P. J. Maas, Roeland B. van Leeuwen, Sylvia Masius-Olthof, Peter Paul G. van Benthem, Tjasse D. Bruintjes

**Affiliations:** ^1^Apeldoorn Dizziness Centre, Gelre Hospital, Apeldoorn, Netherlands; ^2^Department of Otorhinolaryngology and Head and Neck Surgery, Leiden University Medical Center, Leiden, Netherlands

**Keywords:** benign paroxysmal positional vertigo, horizontal, lateral, treatment, geotropic, apogeotropic

## Abstract

**Purpose:** To determine the effectiveness of our treatment protocol for geotropic and apogeotropic horizontal canal benign paroxysmal positional vertigo (h-BPPV).

**Methods:** We retrospectively evaluated patients with newly diagnosed geotropic and apogeotropic h-BPPV who visited our clinic between July 2017 and December 2019. Patients were treated according to our treatment protocol, which was implemented in 2017. Patients with geotropic h-BPPV were preferably treated with the Gufoni maneuver. In patients with apogeotropic h-BPPV we executed the modified Gufoni maneuver to achieve conversion to the geotropic type. We looked at the number of successful treatments and the number of recurrences within 1 year.

**Results:** We included 102 patients with h-BPPV, 62 (61%) of whom were treated for geotropic h-BPPV. The ratio of apogeotropic to geotropic h-BPPV was 0.65. After the first visit, we observed resolution of horizontal canal BPPV in 71 and 63% of the geotropic and the apogeotropic group, respectively. After the second visit, this percentage increased to 92% for geotropic h-BPPV and 78% for apogeotropic h-BPPV. After 1 year of follow-up we determined a recurrence rate of 32 and 24% for the geotropic and apogeotropic group, respectively.

**Conclusion:** With our treatment protocol we managed to achieve high rates of symptom resolution in the geotropic and apogeotropic type of h-BPPV with acceptable recurrence rates. We observed a relatively high ratio of apogeotropic h-BPPV to geotropic h-BPPV.

## Introduction

Benign paroxysmal positional vertigo (BPPV), the most common vestibular type of vertigo, is characterized by sudden and brief spinning sensations initiated by a change of head position (1–3). The majority of BPPV cases involve the posterior semicircular canal (p-BPPV), and in 5% up to 14% the horizontal semicircular canal is affected (4, 5). In 1985, McClure was the first to describe the horizontal canal variant of BPPV (h-BPPV) by reporting seven cases with a history suggesting BPPV (6). On positional testing, all seven patients showed a horizontal nystagmus, beating toward the undermost ear (geotropic), instead of a rotatory upbeat nystagmus, which is typical for p-BPPV. At present, it is thought that in this geotropic variant otoconial debris is located in the posterior arm of the horizontal semicircular canal. In 1995, another variant of h-BPPV was described by Baloh et al. (7). They reported three cases with an apogeotropic nystagmus (nystagmus beating toward the uppermost ear) persisting as long as the lateral position was maintained, which they attributed to debris attached to the cupula (cupulolithiasis). In 1996, Nuti et al. described a transient variant of apogeotropic nystagmus, which they assigned to debris located in the anterior (ampullary) part of the horizontal canal (8). According to the literature, the incidence of the geotropic variant of h-BPPV could be up to three times higher than the incidence of the apogeotropic type (5, 9–11).

Although h-BPPV often occurs spontaneously, it may also arise as a complication after treatment for p-BPPV as a result of canal conversion. This occurs in about 1 to 9% of those treated for p-BPPV with a canalith repositioning maneuver (12–14).

For every case of BPPV, the goal of treatment is to achieve a complete resolution of vertigo by moving the otolithic debris back into the utricle. Unfortunately, the treatment of h-BPPV, especially for the apogeotropic type, has not been as well-established as in p-BPPV. Various maneuvers with variable effectiveness have been described for both types of h-BPPV which could complicate the choice for the optimal treatment. To facilitate treatment of h-BPPV we composed a standardized treatment protocol in 2017 based on the available evidence thus far (15). In the same year, we implemented the protocol in our dizziness centre. The aim of our present research was to determine the effectiveness of this treatment protocol for both the geotropic and the apogeotropic type of h-BPPV.

## Methods

### Data Collection and Definitions

We retrospectively reviewed the medical files of patients who were newly diagnosed with horizontal canal BPPV in our tertiary dizziness clinic between June 2017 and December 2019. We included patients with h-BPPV according to the following criteria: 1. a history of positional vertigo, 2. geotropic or apogeotropic horizontal nystagmus and vertigo elicited by the supine roll test, 3. absence of other disorders that could explain positional vertigo and nystagmus (16). The affected side was determined by assessing the intensity of the nystagmus during the supine roll test. In the geotropic h-BPPV the affected side is the side with the strongest nystagmus, in the apogeotropic type it is the side with the weakest nystagmus. When h-BPPV resulted from a canal conversion after treatment for p-BPPV, the affected side was considered the same as the side with p-BPPV. We collected patient data including sex, age, the number of visits to the dizziness clinic, the type of nystagmus (geotropic or apogeotropic), the affected side, the type of treatment and the outcome of the treatment. Additionally, we recorded whether h-BPPV occurred spontaneously or whether it was the result of a canal conversion after treatment for p-BPPV. A canal conversion was defined as the occurrence of a horizontal nystagmus and dizziness after treatment for p-BPPV without a period of remission.

### Treatment

#### Geotropic h-BPPV

Patients with the geotropic type of h-BPPV were treated with the Gufoni maneuver or the Lempert maneuver. Our preferred treatment is the Gufoni maneuver since it is easy to perform, specifically suitable for older people and it might be more effective than the Lempert maneuver (17). During the execution of the Gufoni maneuver the patient is sitting straight-up and is then quickly tilted to the unaffected side (18). After ~2 min the head is quickly turned 45 degrees down. Eventually, the patient is turned back into the sitting upright position. The Lempert maneuver starts with the patient in a supine position and is followed by three 90 degrees head rotations toward the unaffected side (19). Each head position is maintained for 1 min. Both maneuvers are combined with Vannucchi's forced prolonged position (FPP) to the unaffected side (20).

To determine whether the canalith repositioning maneuver is successful, the patients are scheduled for a diagnostic maneuver 1 week after the initial treatment or for a telephone appointment in case of long travel distance to our clinic. In case a complete resolution of vertigo is not achieved, additional canalith repositioning maneuvers appropriate for the affected canal are performed. Patients are examined again after 1–2 weeks, and treatment is repeated as many times as necessary.

#### Apogeotropic h-BPPV

In patients with apogeotropic h-BPPV our treatment protocol recommends to perform a modified Gufoni maneuver with the intention to achieve conversion to a geotropic nystagmus (17). In the modified Gufoni maneuver the patient is brought to the side-lying position on the affected side (21). After 2 min the head is turned 45 degrees upward (nose up). Eventually, the patient is turned back into the sitting upright position. Fifteen minutes after this maneuver the supine roll test is repeated. In some cases the modified Gufoni maneuver results in an immediate symptom resolution (22). In that case, patients are not subjected to additional maneuvers. If patients show a successful conversion to a geotropic nystagmus, our treatment protocol prescribes to perform the Gufoni maneuver followed by Vannucchi's FPP to the unaffected side. In the event of an ongoing apogeotropic nystagmus, the head-shaking maneuver is executed followed by Vannucchi's FPP to the affected side. Similar to the treatment of geotropic h-BPPV, patients with apogeotropic h-BPPV are examined again after 1–2 weeks, and treatment is repeated as many times as necessary.

#### Undetermined Affected Side

Since the treatment protocol does not provide advice for patients in whom the affected side could not be determined, these patients were solely treated with a head-shaking maneuver. The head-shaking maneuver was performed with the patient sitting in the upright position with the head bent 30 degrees forward in the plane of the horizontal semicircular canal. Subsequently, we shook the head sideways in a sinusoidal movement with a velocity of 2Hz for ~15–30 s.

### Treatment Outcomes

Treatment for horizontal canal BPPV was considered successful if the follow-up supine roll test provoked neither dizziness nor horizontal nystagmus. If patients were successfully treated for h-BPPV but complaints persisted and the provoking tests showed a posterior canal BPPV, we considered this as a persisting BPPV and treated them with the appropriate maneuver. Overall treatment was considered successful if the follow-up diagnostic maneuvers showed neither dizziness nor horizontal or posterior canal nystagmus. If patients were contacted by telephone, treatment was considered successful if they reported no complaints. Recurrence was defined as the appearance of ipsi- or contralateral, geotropic or apogeotropic horizontal or posterior canal BPPV according to the accepted international criteria within 1 year after successful treatment (16).

### Data Analysis

Differences between groups were tested with the Fisher's exact test for categorical data and the Mann-Whitney U test for non-parametric continuous data. A *p* < 0.05 was considered statistically significant. SPSS version 25.0 was used for the analyses.

### Ethical Approval and Informed Consent

The study was approved by the clinical research ethics committee of our hospital and performed in accordance with the ethical standards as laid down in the 1964 Declaration of Helskinki and its later amendments up to 2013 (23). Informed consent was not obtained from the participants since all data analyzed were collected as part of routine diagnosis and treatment.

## Results

We identified 102 patients with h-BPPV who had been referred to our dizziness clinic between July 2017 and December 2019. A total of 62 (61%) patients had a geotropic h-BPPV and 40 (39%) had the apogeotropic type. This resulted in a ratio of apogeotropic to geotropic h-BPPV of 0.65. We observed no significant differences between the two groups regarding sex, median age and the number of patients contacted by telephone (Table 1). Canal conversion after treatment for p-BPPV was more often the cause of h-BPPV in patients with the geotropic variant than in patients with the apogeotropic variant (*p* = 0.03).

**Table 1:** Baseline characteristics of patients with geotropic h-BPPV and apogeotropic h-BPPV.

**Characteristics**	**Geotropic h-BPPV**	**Apogeotropic h-BPPV**	***p-*value**
	***(n = 62)***	***(n = 40)***	
Female sex (%)	47 (76%)	25 (63%)	0.2
Median age (IQR)	69 years (58–78)	70 years (63–78)	0.4
Telephone contact	9 (15%)	4 (10%)	0.8
Canal conversion caused by treatment of p-BPPV (%)	14 (23%)	2 (5%)	0.03

*IQR, interquartile range*.

### Geotropic Nystagmus

**Figure 1:**
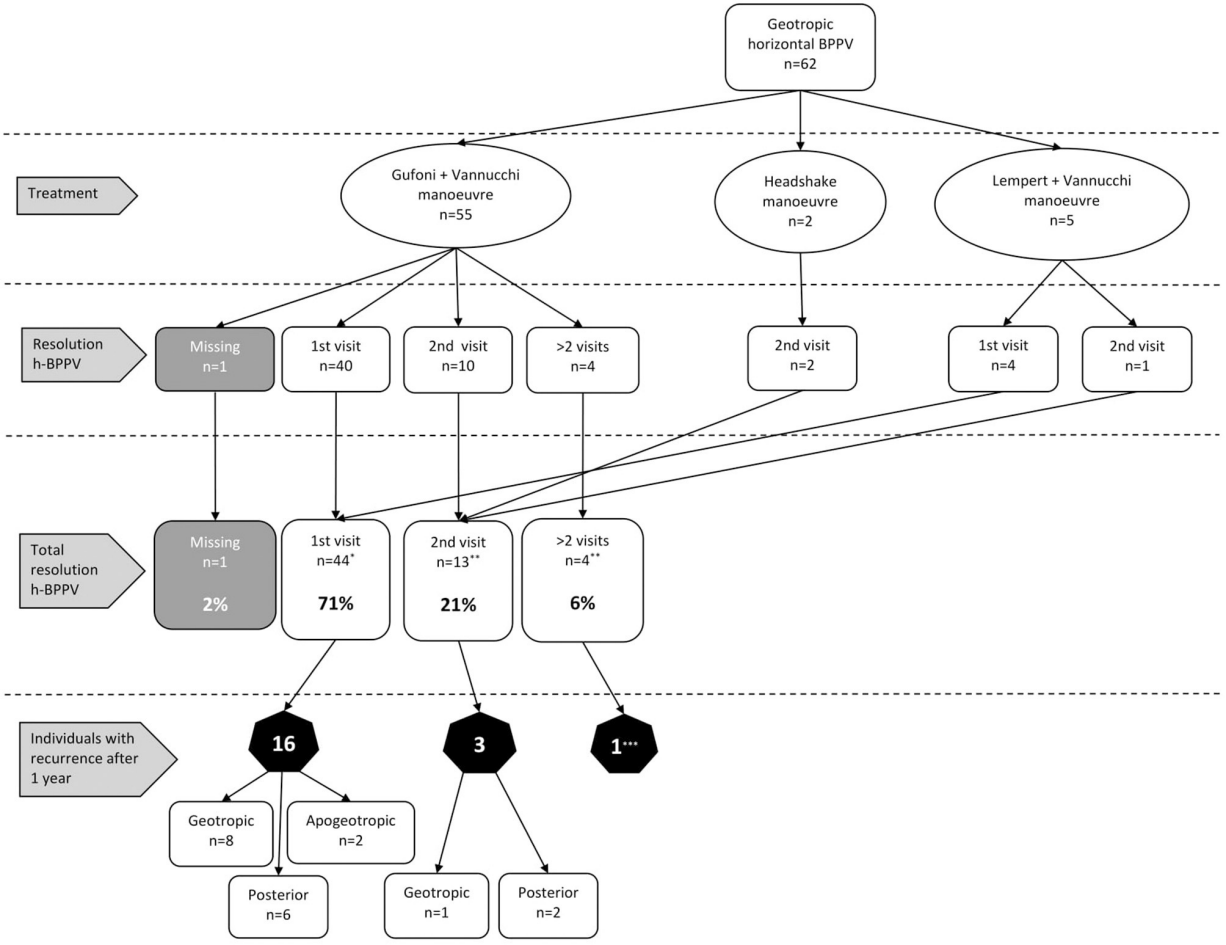
Results of our treatment protocol and the number of recurrences for geotropic h-BPPV. (*) 14 patients received additional treatment for posterior canal BPPV. (**) 3 patients received additional treatment for posterior canal BPPV. (***) 1 patient with a suspected recurrence when contacted by telephone. The type of recurrence was not documented since this patient decided to continue treatment elsewhere.

A total of 62 patients were diagnosed with geotropic h-BPPV (Figure 1). After the affected side was identified, 55 patients were treated with the combination of a Gufoni maneuver followed by Vannucchi's FPP and five patients were treated with the Lempert maneuver followed by Vannucchi's FPP. In two cases the headshake maneuver was executed since the affected side could not be determined. After the first visit, we observed resolution of horizontal canal BPPV in 71% (*n* = 44). This percentage increased to 92% (*n* = 57) after the second visit. Only four (6%) patients needed more than two visits. One of them withdrew from repositioning maneuvers in our dizziness clinic after the fourth visit and reported no improvement of BPPV symptoms after various treatments elsewhere. In 20 out of 62 patients (32%) with geotropic h-BPPV a canal switch to p-BPPV occurred after successful treatment for h-BPPV. Appropriate additional repositioning maneuvers were performed and symptom resolution was achieved in all 20 patients. In 20 patients (32%) we detected at least one recurrence. The median time for recurrences to occur was 111 days (IQR: 63–172 days).

### Apogeotropic Nystagmus

**Figure 2:**
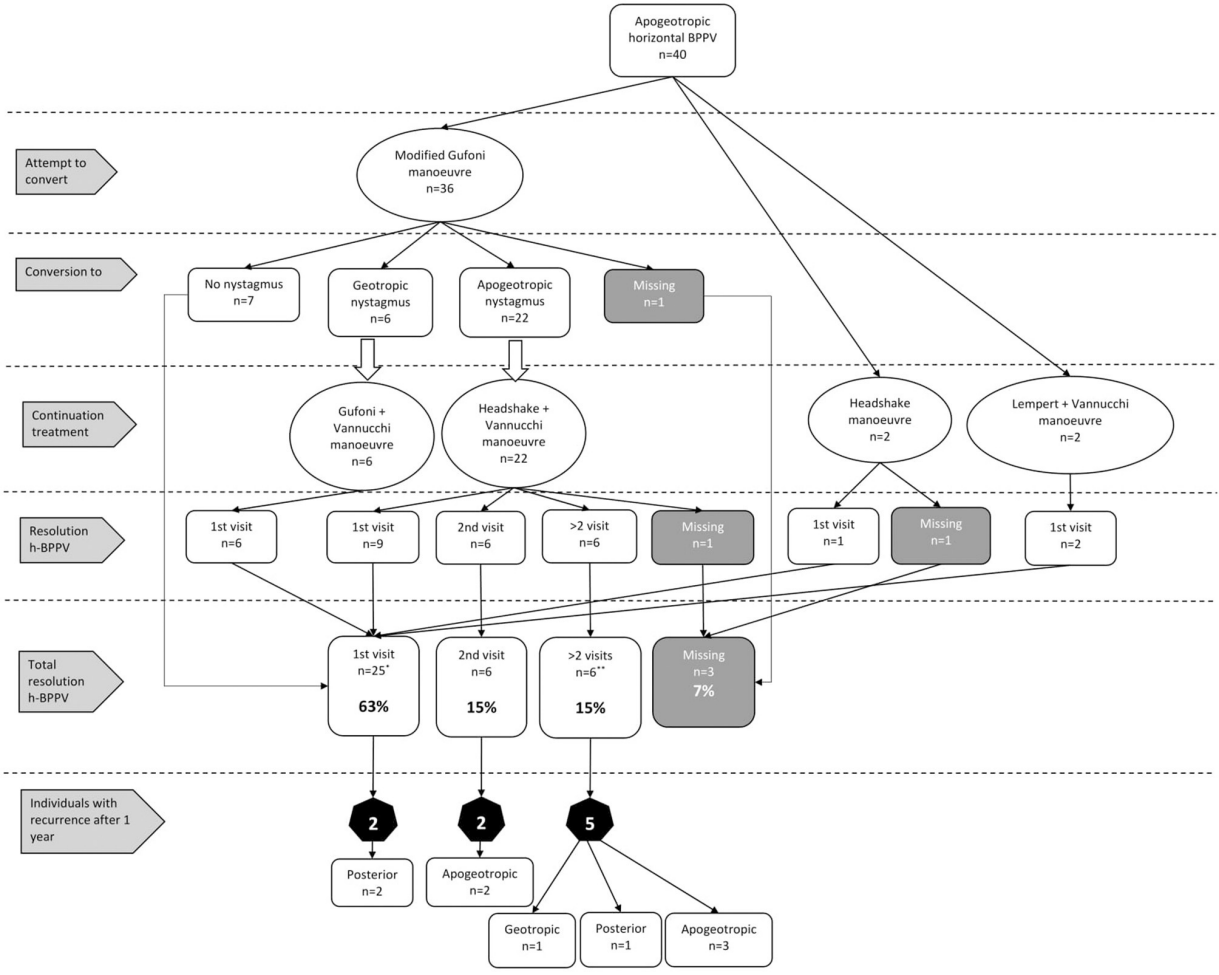
Results of our treatment protocol and the number of recurrences for apogeotropic h-BPPV. (*) 5 patients received additional treatment for posterior canal BPPV. (**) 1 patient received additional treatment for posterior canal BPPV.

We treated a total of 40 patients with apogeotropic h-BPPV (Figure 2). Thirty-six (90%) patients were treated with a modified Gufoni maneuver in order to convert the nystagmus in line with our treatment protocol. In two patients the Lempert maneuver was erroneously performed and two patients were treated with the headshake maneuver because the affected side could not be determined.

Treatment with the modified Gufoni maneuver resulted in a complete resolution of horizontal canal BPPV in 19% (*n* = 7) and in a conversion to geotropic nystagmus in 17% (*n* = 6). In the majority of patients (*n* = 22; 61%) an apogeotropic nystagmus persisted. In one patient data about conversion were missing.

Patients with a geotropic nystagmus after conversion were all treated with the (regular) Gufoni maneuver, followed by Vannucchi's FPP. All six patients showed complete resolution of horizontal canal BPPV after this treatment.

The 22 patients with a persisting apogeotropic nystagmus after the conversion attempt were treated with a headshake maneuver combined with Vannucchi's FPP. After this treatment, almost half of these patients (*n* = 9; 41%) showed resolution of horizontal canal BPPV. In six patients a resolution was observed after two visits and in the remaining six patient symptom resolution was observed after more than two visits. For one patient data about symptom resolution were missing.

Two patients were treated with a headshake maneuver combined with Vannucchi's FPP, this resulted in resolution of horizontal canal BPPV after the first visit in one patient. For the other patient data were missing. The Lempert maneuver combined with Vannucchi's FPP was also performed in two patients. This resulted in resolution of horizontal canal BPPV in both patients after the first visit.

Of the 40 patients, a total of 25 (63%) showed resolution of horizontal nystagmus after the first visit. This percentage increased to 78% (*n* = 31) after the second visit. In six out of 40 patients (15%) with apogeotropic h-BPPV a canal switch to p-BPPV occurred after successful treatment for h-BPPV. Appropriate additional repositioning maneuvers were performed and symptom resolution was achieved in all six patients. Nine patients (24%) showed at least one recurrence of horizontal or posterior BPPV. Recurrences occurred after a median time of 87 days (IQR: 23–165 days).

## Discussion

This retrospective study demonstrates the outcomes of treatment in 102 patients with either geotropic or apogeotropic h-BPPV in our tertiary dizziness clinic. After implementation of our treatment protocol in 2017, we aimed to determine the effectiveness of this protocol. The study shows that the first treatment resulted in resolution of horizontal canal BPPV in 71 and 63% of patients in the geotropic and apogeotropic group, respectively. After the second maneuver this percentage increased to 92 and 78%, respectively. The total resolution of both horizontal and posterior canal BPPV was slightly lower. This is because a substantial part of the patients developed a posterior canal BPPV after a resolution of horizontal canal BPPV which might be due to multiple canal involvement or canal switch from h-BPPV to p-BPPV (12, 24).

The treatment results in patients with geotropic h-BPPV are generally in line with the literature, with published success rates varying from 61% up to 90% after the second treatment (9, 11, 25).

The outcomes in patients with apogeotropic h-BPPV are not entirely comparable to those of other studies, since there are various therapy options for the apogeotropic type. Although most studies agree with the stepwise procedure of conversion followed by treatment, methods differ across studies. Methods for conversion vary from a Pagnini-McClure maneuvers to a head-shaking maneuver to a modified Gufoni maneuver (9–11, 26).

Kim et al. (26) and Appiani et al. (21) also used the modified Gufoni maneuver in order to convert the nystagmus from apogeotropic into geotropic. In line with our results, Kim et al. reported a complete resolution of 60% after the first maneuver and of 73% after the second maneuver (26). Appiani et al. successfully performed the modified Gufoni maneuver in eight patients, all of whom showed a conversion from apogeotropic to geotropic h-BPPV after the first maneuver (21). After treating the geotropic variant, all patients were cured from h-BPPV.

The higher conversion rate achieved by Appiani et al. (21) might have been due to differences in patient characteristics. Appiani et al. (21) treated patients who had reported complaints since ~7 days, whereas we treated patients who had been referred for a second or third opinion. Although we did not document the time of onset of complaints in our study population, it is likely that it was more than 1 week, which might have resulted in a population with a more intractable type of BPPV, as the natural course of h-BPPV appears to be short (27, 28).

Within 1 year, we found recurrences in 32% of the patients in the geotropic and in 24% of apogeotropic group. Our recurrence rates are slightly higher than reported in the literature (29–32). However, some studies had a shorter follow-up time and others might not have included the posterior canal type of BPPV as a recurrence. Besides, the patients treated in our dizziness clinic are likely to have more intractable BPPV which could explain the somewhat higher recurrence rates. In the apogeotropic group, most recurrences occurred in patients who needed multiple treatments. On the contrary, the geotropic group showed most recurrences in patients who showed a resolution of h-BPPV after the first treatment. For this reason, it is important to re-examine or call patients who have been treated successfully to establish whether complaints recurred. Furthermore, it could be valuable to offer patients easy access for re-examination in case complaints reoccur. In our dizziness clinic, we have good experiences with allowing patients easy access for re-examination for a period of 3 years after the first treatment.

We found a ratio of apogeotropic h-BPPV to geotropic h-BPPV of 0.65. This is higher than the ratio generally reported in the literature (5, 9–11). Only Casani et al. reported a similar distribution with 38% of apogeotropic cases and 62% of geotropic cases (11). This higher ratio may result from the fact that our study was performed in a tertiary referral centre. Patients with apogeotropic h-BPPV are more often referred to a tertiary clinic, as the identification of the affected side and treatment is expected to be more difficult.

Furthermore, in 16 patients, h-BPPV was the result of a canal conversion after treatment for p-BPPV. In 14 cases this resulted in a geotropic h-BPPV, and in two cases it caused an apogeotropic h-BPPV. These outcomes are in line with data reported in previous studies (12, 13). Since a canal conversion occurs from a re-entry of otolithic debris from the posterior canal into the horizontal canal via the utricle, it is reasonable to assume that the debris floats into the posterior part of the horizontal semicircular canal, which causes a geotropic h-BPPV rather than an apogeotropic h-BPPV (14).

As pointed out before, the number of patients who were successfully converted from an apogeotropic h-BPPV to a geotropic h-BPPV or to a resolution of h-BPPV was limited (36%). This might be due to the exact location of the otolithic debris. If the debris is located in the anterior part of the horizontal semicircular canal, conversion might be successful. However, it might be more complicated to convert debris attached to the cupula. It is difficult to exactly localize the otolithic debris since it may be situated in the anterior part of the semicircular canal or it could be attached to the utricular or canal-side of the cupula.

New techniques to avoid difficulties in localizing the otolithic debris in apogeotropic h-BPPV were recently introduced and analyzed. Zuma et al. described a repositioning maneuver comparable to the modified Gufoni maneuver for apogeotropic cupulolithiasis and canalolithiasis (33). They added two more steps which should detach the debris from the utricular side of the cupula. Retrospectively, the authors found a resolution rate of 100% in 8 patients with apogeotropic h-BPPV.

A second repositioning maneuver, the cupulolith repositioning maneuver (CuRM) treating all types of apogeotropic h-BPPV was described by Kim et al. (34). In this repositioning treatment simultaneous oscillation is used to detach the otolithic debris from both sides of the cupula. The effectiveness of this treatment was prospectively evaluated in a randomized controlled trial by Kong et al. (35). Although there were no significant differences in the resolution rates between the CuRM and the head-shake maneuver, the authors found a success rate for the CuRM of up to 90% after one treatment.

The first and main limitation of our study lies in its retrospective nature, which resulted in some missing data. In case of missing data, we attempted to contact patients by telephone to determine whether treatment had been successful and to ask about potential recurring symptoms. Second is the inconsistency in follow-up. Most patients (85%) were followed up by a diagnostic maneuvers but some patients were followed up by telephone.

To better establish the effectiveness of our treatment protocol for geotropic and apogeotropic h-BPPV and to compare it to other canalith repositioning maneuvers, future prospective research should be undertaken.

This study shows that our treatment protocol for horizontal canal BPPV leads to high rates of symptom resolution in patients with geotropic h-BPPV as well as in patients with apogeotropic h-BPPV. For a tertiary dizziness clinic, it is important to be aware of the relatively high numbers of patients with apogeotropic h-BPPV since it requires a more comprehensive treatment procedure than geotropic h-BPPV. After 1 year of follow-up, our study shows fairly high recurrence rates which emphasizes the importance of follow-up and re-examination in this group of patients. Continued efforts are needed to determine the optimal treatment for both geotropic and apogeotropic h-BPPV.

## Data Availability Statement

The original contributions presented in the study are included in the article/supplementary material, further inquiries can be directed to the corresponding author/s.

## Ethics Statement

The studies involving human participants were reviewed and approved by the Local Review Board of Gelre Hospital. Written informed consent for participation was not required for this study in accordance with the national legislation and the institutional requirements.

## Author Contributions

BM, SM-O, and TB: research idea. BM and SM-O: designed the study. BM: analyzed the data and wrote the manuscript. RL, SM-O, PB, and TB revised the manuscript. All authors contributed to the article and approved the submitted version.

## Conflict of Interest

The authors declare that the research was conducted in the absence of any commercial or financial relationships that could be construed as a potential conflict of interest.
